# Supplementary material for: Virus Behavior after UV254 Treatment of Materials with Different Surface Properties
Source: Microorganisms. 2023 Aug 25;11(9):2157. doi: 10.3390/microorganisms11092157 (PMC10535119; doi:10.3390/microorganisms11092157)
Supplement: Supplementary file 1 [file microorganisms-11-02157-s001.zip › microorganisms-2517968-supplementary.pdf]

Supplemental Information for *Virus Behavior after UV_{254} Treatment of Materials with Different Surface Properties*

Castine Bernardy, James Malley

Department of Civil and Environmental Engineering, College of Engineering & Physical Sciences, University of New Hampshire, Durham, NH 03824, USA

Figures

The data below display the results of the swabbing and rinsing experiments on the aluminum disks.

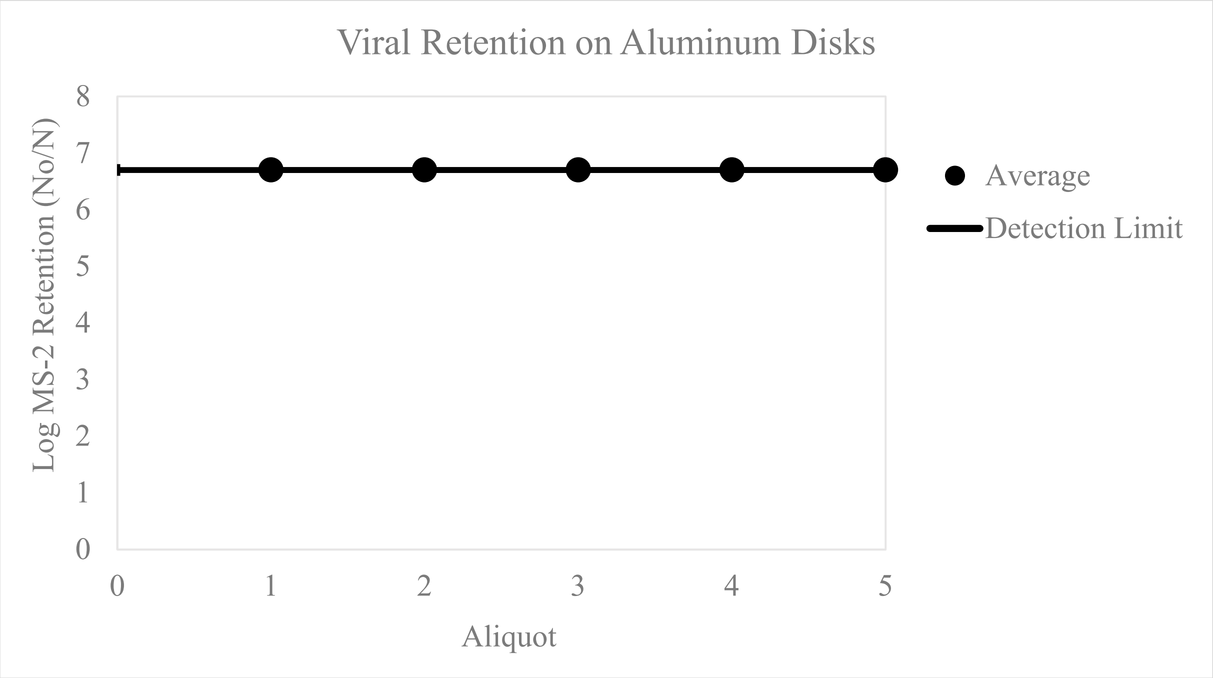

Figure S1 displays the results of the aluminum swabbing and rinsing experiments.

Figure S1 displays the results of the aluminum swabbing and rinsing experiments. These experiments agitated the aluminum surface repeatedly with a cotton swab, in an attempt to recover MS-2 bacteriophage. MS-2 was not recovered from the surface in any of the rinsing aliquots. The figure displays the MS-2 retention from each aliquot at the detection limit.

Statistical Data: Equivalency Testing

Figures S2-S4 below display equivalence testing outputs created in JMP. These data compare the k values (slopes) of the UV_{254} dose response curves for ceramic, Formica laminate, PTFE and stainless steel. The goal of equivalency testing is to determine if the variation in slopes is statistically relevant to the UV disinfection field.

Equivalence Tests with Unequal Variances

Lower Bound = -0.0032, Upper Bound = 0.0032, α level= 0.05

Equivalence TOST Tests

Surface	-Surface	Difference	Std Error of Difference	Lower Bound t Ratio	Upper Bound t Ratio	Lower Bound p-Value	Upper Bound p-Value	Max p-Value	Lower 90%	Upper 90%
Ceramic	Formica Laminate	-0.007400	0.0044157	-0.9512	-2.4005	0.8045	0.0344*	0.8045	-0.016586	0.001786
Ceramic	PTFE	-0.181100	0.0120305	-14.7874	-15.3194	0.9999	<.0001*	0.9999	-0.207315	-0.154885
Ceramic	Stainless Steel	-0.002875	0.0042262	0.0769	-1.4375	0.4713	0.1136	0.4713	-0.012011	0.006261
Formica Laminate	PTFE	-0.173700	0.0115264	-14.7921	-15.3474	0.9998	0.0002*	0.9998	-0.200220	-0.147180
Formica Laminate	Stainless Steel	0.004525	0.0024466	3.1575	0.5416	0.0108*	0.6955	0.6955	-0.000294	0.009344
PTFE	Stainless Steel	0.178225	0.0114552	15.8378	15.2791	0.0002*	0.9997	0.9997	0.151621	0.204829

Equivalence Tests Scatterplot

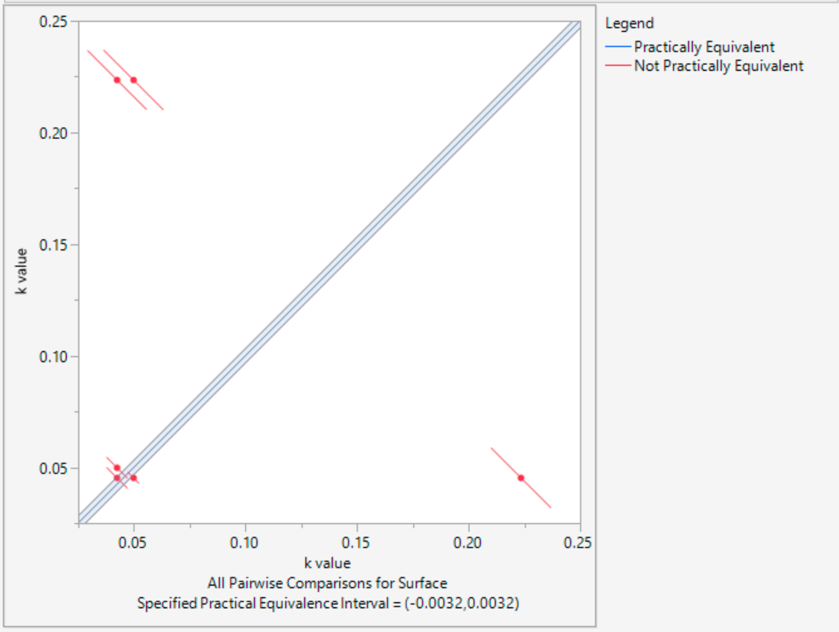

Figure S2 displays the JMP output for equivalency testing conducted on the slopes of the dose response curves for each surface (ceramic, Formica laminate, PTFE and stainless steel). The "practically zero" value for this output was set at 0.0032.

Figure S2 displays the equivalence testing output created in JMP. The essentially zero" value for this output was set at 0.0032. This value was taken from *Fluence (UV Dose) Required to Achieve Incremental Inactivation of Bacteria, Protozoa, Viruses and Algae* (2016) by Malayeri et al [13]. This publication tabulated the works of previous researchers, reporting on the log inactivation levels of MS-2 as a function of UV dose. The difference in k values between the upper and lower standard deviation bounds was used as the practically zero value for this analysis.

The JMP output displays that the slopes of the UV dose response curves for each surface are not practically equivalent. This can be observed visually by the red markers, as a portion of all of them lies in the not practically equivalent zone of the graph. In addition, to be practically equivalent, the *p-values* reported for the lower and upper bounds have to be below 0.05. Therefore, indicating the surfaces k values are not practically equivalent.

Equivalence Tests with Unequal Variances

Lower Bound = -0.0102, Upper Bound = 0.0102, α level= 0.05

Equivalence TOST Tests

Surface	-Surface	Difference	Std Error of Difference	Lower Bound t Ratio	Upper Bound t Ratio	Lower Bound p-Value	Upper Bound p-Value	Max p-Value	Lower 90%	Upper 90%
Ceramic	Formica Laminate	-0.007400	0.0044157	0.6341	-3.9858	0.2788	0.0068*	0.2788	-0.016586	0.001786
Ceramic	PTFE	-0.181100	0.0120305	-14.2055	-15.9012	0.9999	<.0001*	0.9999	-0.207315	-0.154885
Ceramic	Stainless Steel	-0.002875	0.0042262	1.7332	-3.0938	0.0808	0.0194*	0.0808	-0.012011	0.006261
Formica Laminate	PTFE	-0.173700	0.0115264	-14.1848	-15.9547	0.9997	0.0002*	0.9997	-0.200220	-0.147180
Formica Laminate	Stainless Steel	0.004525	0.0024466	6.0187	-2.3196	0.0006*	0.0313*	0.0313*	-0.000294	0.009344
PTFE	Stainless Steel	0.178225	0.0114552	16.4489	14.6681	0.0002*	0.9997	0.9997	0.151621	0.204829

Equivalence Tests Scatterplot

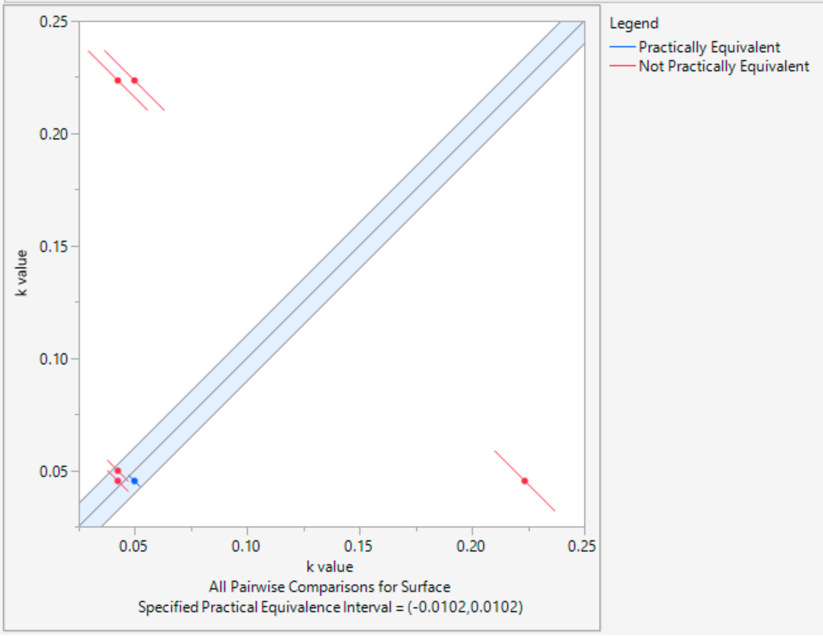

Figure S3 displays the JMP output for equivalency testing conducted on the slopes of the dose response curves for each surface (aluminum, ceramic, Formica laminate, PTFE and stainless steel). The "practically zero" value for this output was set at 0.0102.

Figure S3 displays the equivalence testing output created in JMP. The essentially zero value for this output was set at 0.0102. This value was taken from the Ultraviolet Germicidal Irradiance Handbook (2009) by Kowalski [2]. The handbook lists k values for MS-2 bacteriophage inactivation from a large body of research. Only the data for water UV_{254} disinfection was tabulated, to avoid skewing the values by mixing mediums. The standard deviation for these reported data was used as the essentially zero value.

The JMP output displays that the slopes of the UV dose response curves for Formica laminate and stainless steel surface are practically equivalent. This can be observed visually by the blue markers, as their entirety of all lies in the practically equivalent zone of the graph. In addition, to be practically equivalent, the *p-values* reported for the lower and upper bounds have to be below 0.05. Therefore, indicating the Formica laminate and stainless steel inactivation rate constants (k values) are practically equivalent. Practical equivalence was not determined for the other surfaces.

Equivalence Tests with Unequal Variances

Lower Bound = -0.0065, Upper Bound = 0.0065, α level= 0.05

Equivalence TOST Tests

Surface	-Surface	Difference	Std Error of Difference	Lower Bound t Ratio	Upper Bound t Ratio	Lower Bound p-Value	Upper Bound p-Value	Max p-Value	Lower 90%	Upper 90%
Ceramic	Formica Laminate	-0.007400	0.0044157	-0.2038	-3.1479	0.5762	0.0153*	0.5762	-0.016586	0.001786
Ceramic	PTFE	-0.181100	0.0120305	-14.5131	-15.5937	0.9999	<.0001*	0.9999	-0.207315	-0.154885
Ceramic	Stainless Steel	-0.002875	0.0042262	0.8577	-2.2183	0.2208	0.0470*	0.2208	-0.012011	0.006261
Formica Laminate	PTFE	-0.173700	0.0115264	-14.5058	-15.6337	0.9997	0.0002*	0.9997	-0.200220	-0.147180
Formica Laminate	Stainless Steel	0.004525	0.0024466	4.5063	-0.8073	0.0024*	0.2263	0.2263	-0.000294	0.009344
PTFE	Stainless Steel	0.178225	0.0114552	16.1259	14.9911	0.0002*	0.9997	0.9997	0.151621	0.204829

Equivalence Tests Scatterplot

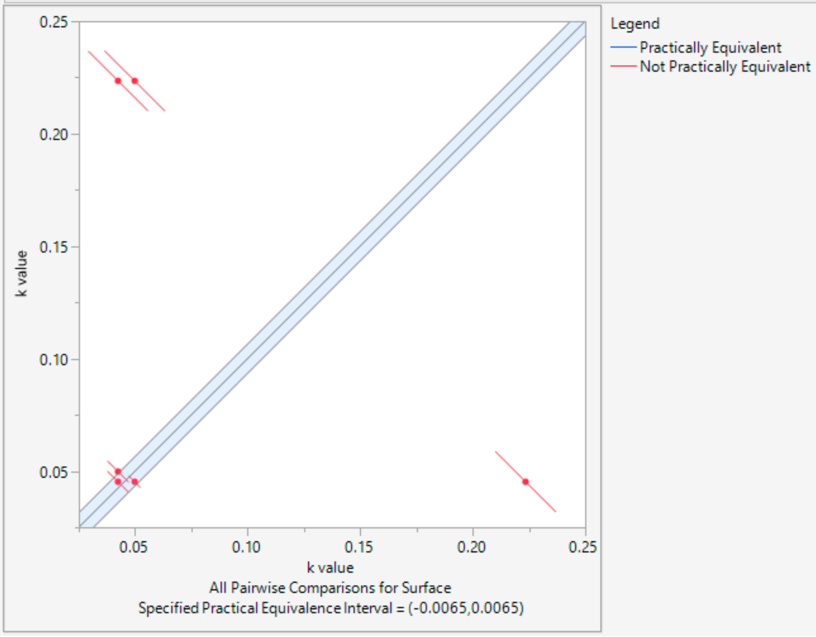

Figure S4 displays the JMP output for equivalency testing conducted on the slopes of the dose response curves for each surface (aluminum, ceramic, Formica laminate, PTFE and stainless steel). The "practically zero" value for this output was set at 0.0065.

Figure S4 displays the equivalence testing output created in JMP. The essentially zero value for this output was set at 0.0065. This value was taken from *Ultraviolet disinfection guidelines for drinking water and water reuse* (2012) by Emerick et al. [22]. This publication provided a MS-2 dose response curve for water disinfection tabulated from a large group of reputable researchers. The difference in k values between the upper and lower standard deviation bounds was used as the practically zero value for this analysis.

The JMP output displays that the slopes of the UV dose response curves for each surface are not practically equivalent. This can be observed visually by the red markers, as a portion of all of them lies in the not practically equivalent zone of the graph. In addition, to be practically equivalent, the *p-values* reported for the lower and upper bounds have to be below 0.05. Therefore, indicating the surfaces k values are not practically equivalent.

As shown in Figures S2-S4, equivalency testing was conducted three times, each time using a different method to calculate the essentially zero input value. Although, the results of

each test were unchanged each run, providing confidence that the slopes of the UV dose response curves for each surface are not practically equivalent.

Surface Characteristics

The surface characteristics (displayed in Table 3) are shown below in Figures S5-S9. These figures display the relationship between the surface characteristics analyzed for this research.

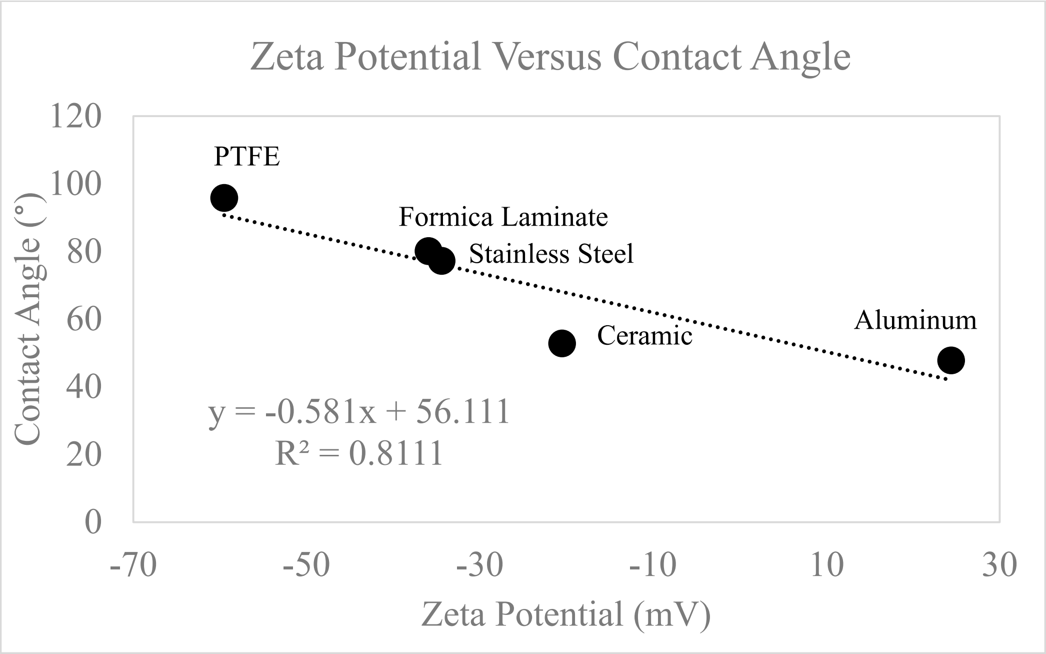

Figure S5 displays zeta potential versus contact angle for the five surfaces analyzed for this research. Zeta potential (mV) is shown on the x axis. Contact angle (°) is displayed on the y axis.

Figure S5 displays zeta potential versus contact angle for aluminum, ceramic, Formica laminate, PTFE, and stainless steel. These data display an inverse, linear relationship, such that as contact angle (°) decreases, zeta potential (mV) increases.

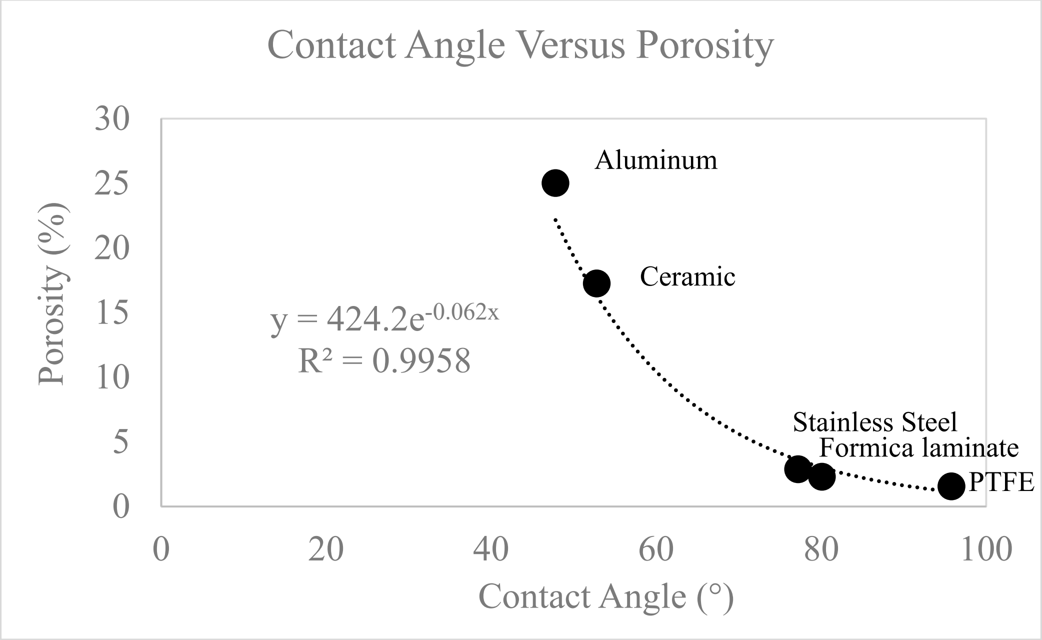

Figure S6 displays contact angle versus porosity for the five surfaces analyzed for this research. Contact angle (°) is shown on the x axis. Percent porosity, measured with SEM, is shown on the y axis.

Figure S6 displays contact angle versus SEM porosity for aluminum, ceramic, Formica laminate, PTFE, and stainless steel. These data display an inverse, exponential relationship, such that as contact angle (°) increases, percent porosity decreases.

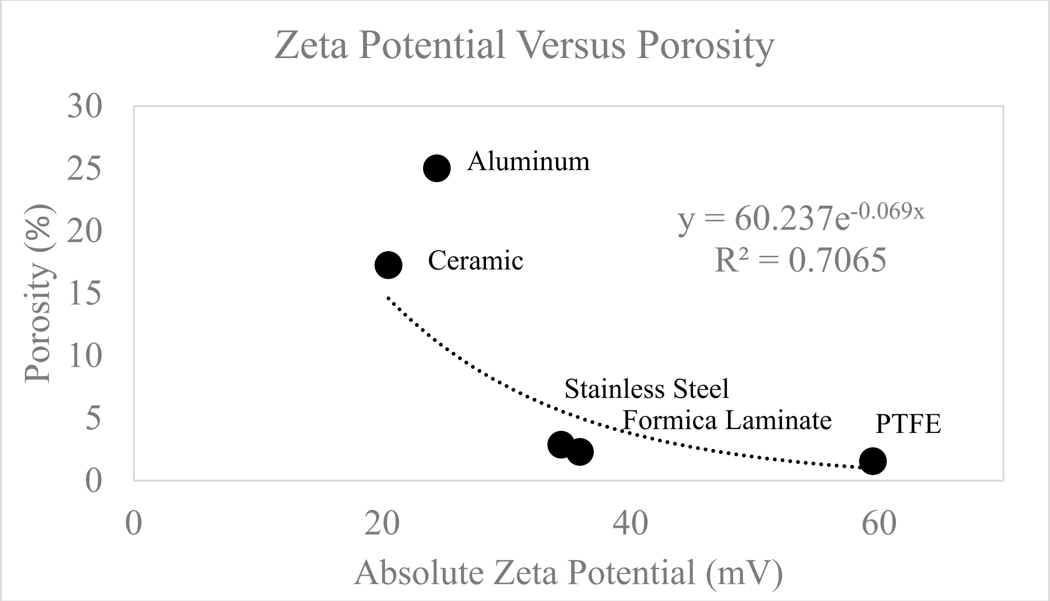

Figure S7 displays absolute zeta potential versus contact angle for the five surfaces analyzed for this research. Zeta potential (mV) is shown on the x axis. Porosity (%) is displayed on the y axis.

Figure S7 displays absolute zeta potential versus contact angle for aluminum, ceramic, Formica laminate, PTFE, and stainless steel. These data display an inverse, non- linear relationship, such that as porosity (%) decreases, zeta potential (mV) increases.

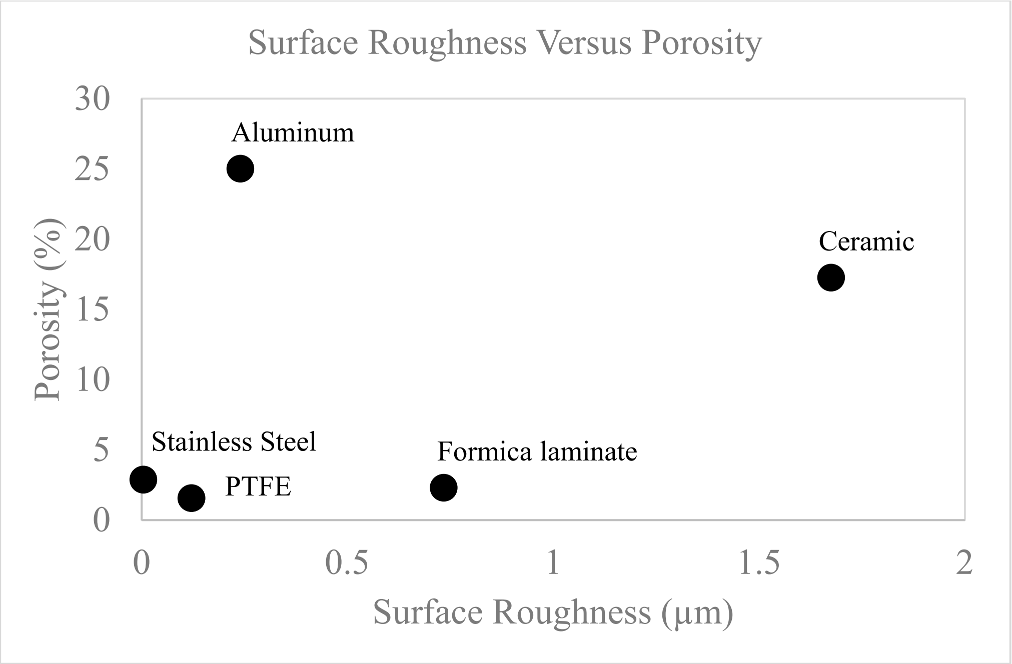

Figure S8 displays surface roughness versus porosity for the five surfaces analyzed for this research. Surface roughness was measured in µm and is shown on the x axis. Percent porosity, measured with SEM, is shown on the y axis.

Figure S8 displays surface roughness (µm) versus percent porosity for aluminum, ceramic, Formica laminate, PTFE, and stainless steel. This figure suggests that a correlation between porosity and surface roughness does not exist for these surfaces.

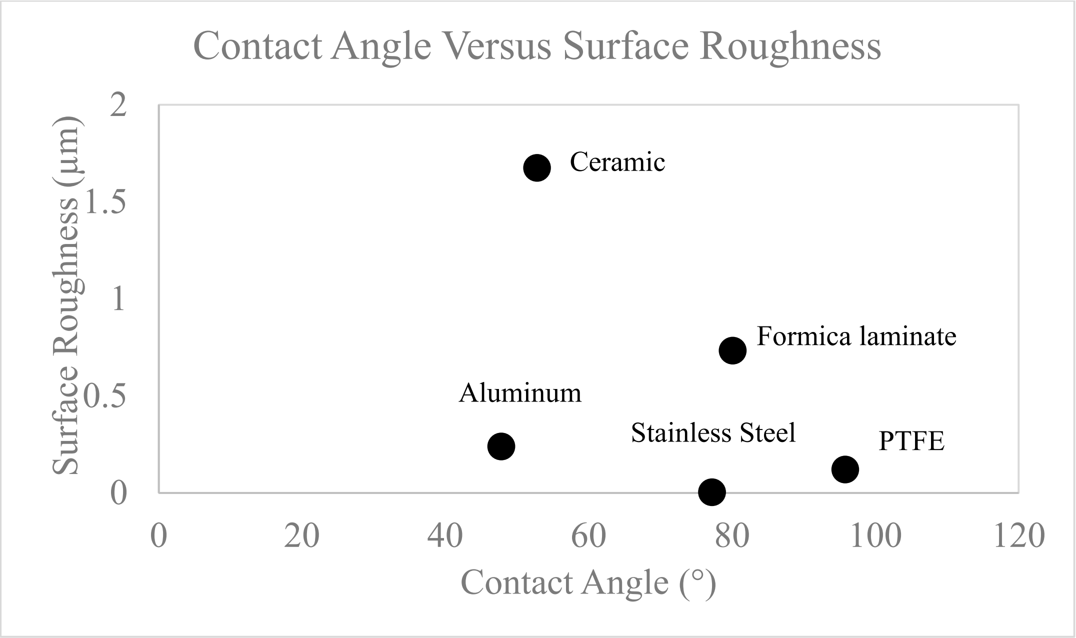

Figure S9 displays contact angle versus surface roughness for the five surfaces analyzed for this research. Contact angle (°) is shown on the x axis. Surface roughness was reported in µm and is displayed on the y axis.

Figure S9 displays contact angle versus surface roughness for aluminum, ceramic, Formica laminate, PTFE, and stainless steel. These data display an inverse, linear relationship, for ceramic, Formica laminate and PTFE, such that as contact angle increases, surface roughness decreases. Aluminum and stainless steel do not follow this relationship.

Tables

Contact Angle Data

The following data (Table S1) were collected on the contact angle of each surface.

Table S1
Average Contact Angle For Each Surface

Surface	Contact Angle	Standard Deviation
Aluminum	47.8	9.2
Ceramic	52.8	7.7
Formica Laminate	80.1	2.5
PTFE	95.8	3.6
Stainless Steel	77.2	4.4

Table S1 displays the average and standard deviation of the contact angles for each surface.

Table S1 above displays the average and standard deviation of the contact angles collected for aluminum, ceramic, Formica laminate, PTFE and stainless steel. These data were collected with an optical tensiometer and are shown in Figure 7 of the text.

Porosity data

The following data (Table S2) were collected on the porosity of each surface.

Table S2
Porosity for Each Surface Type

Surface	Porosity (%)
Aluminum	25.02
Ceramic	17.26
Formica Laminate	2.31
PTFE	1.56
Stainless Steel	2.88

Table S2 displays the porosity for each surface.

Table S2 above displays the calculated porosity of aluminum, ceramic, Formica laminate, PTFE and stainless steel. These data were collected by analyzing magnified SEM images with NIS-Elements software. These data are displayed in Figure 10 of the text.
